# Supplementary material for: Outcomes of excessive alcohol drinkers without baseline evidence of chronic liver disease after 15 years follow-up: Heavy burden of cancer and liver disease mortality
Source: PLoS One. 2021 May 25;16(5):e0252218. doi: 10.1371/journal.pone.0252218 (PMC8148371; doi:10.1371/journal.pone.0252218)
Supplement: Attachment — Submitted filename: RESPONSE TO THE REVIEWERS - 2021.05.09.docx [file pone.0252218.s002.docx]

**RESPONSE TO THE REVIEWERS**

We thank the editor and the reviewers for the constructive critiques and for the opportunity to improve our manuscript. We hope the reviewers now find it worthy for publication. It follows the point-by-point response to the editors and reviewers comments.

RESPONSE TO REVIEWER 3

*Thank you for the revised version of this manuscript.*

*I have just a few additional suggestions and comments:*

*General:
• Please throughout the manuscript state both absolute numbers and proportions. The cohort is relatively small, so the sole reporting of percentages gives a wrong impression.*

R: It was changed accordingly.

• *Language: English editing is still needed. There remain grammar errors and sentence structures, which gives a poor impression, and at certain places makes the text difficult to even understand.*

R: The English was carefully edited.

*• LC is not a common abbreviation; I suggest to write liver cirrhosis in full throughout.*

R: Thank you for the suggestion. It was changed accordingly.

*Results:
• There is a lack of time to progression for all outcomes, except for the Kaplan Meier plots. This hinders accurate interpretation, and should be stated in the text.*

R: It was stated in the text: “**In order to allow for an analysis of time to progression, Kaplan Meyer curves were computed**.” We also added in the discussion: “**Lastly, the analysis of the effect of variables and confounders on outcomes has the risk of bias by the lack of control to time to progression, which was taken into account in the evaluation of the survival curves.**”

*• Figure 1: The ROC analysis is not the correct method to assess prognostic accuracy as it is not time-dependent. Should be replaced with Harrell’s C or time-dependent ROC curves.*

R: We agree that ROC analysis was not adequate to evaluate the abstinence time, as such we removed it in the previous revision of the manuscript.

*Discussion:
• Based on the low event rate and relatively high survival for this type of patient I still suspect a high degree of survivors bias. The authors correctly state: “The first striking finding, from this cohort, is that patients who have no evidence of CLD despite being heavy-drinkers for 25 years are still at increased risk for CLD”. I suggest to more clearly acknowledge and discuss the limitation of survivors bias and that this is not an inception cohort. Rather, included participants have already survived >20 years of heavy drinking without developing evidence of chronic liver disease and this population is therefore selected to be less likely to develop liver disease during the next 14 years, than the average patients with heavy drinking.*

*This is the main interest of this cohort. Albeit it is a highly selected cohort of patients less likely to develop CLD, since they already survived more than 20 years of heavy drinking without developing evidence of CLD, 6% still progresses. The risk of progression is not related with past alcohol consumption, rather than on ongoing alcohol intake.*

R: We changed the sentence to: “The first striking finding, from this cohort, **is that patients who survived up to 25 years of heavy-drinking without developing evidence of CLD, and hence who were more likely resistant to the dismal effects of alcohol on the liver**, are still at increased risk for CLD.”

*• Regarding excess cancer risk due to combined alcohol and smoking. The sentence “This population should probably be submitted to a personalized screening program.” is unsubstantiated as this cohort is not suited to investigate such topic.*

R: We agree with the reviewer, and as such, we changed the sentence to: “**Further studies should address if this population should be submitted to a personalized screening program.**”

*• Similarly the discussion about beer drinking having a possible protective effect is purely speculative and this study is not powered or designed to make such assumptions. The effect of beer may also be that it is the type of beverage containing the lowest percentage of alcohol; or just a common type I error.*

R: We removed the paragraph regarding the discussion about the possible protective effect of beer drinking.

*• The discussion of lack of liver biopsy may be replaced with lack of accurate confirmatory tests – LB or LSM – to ensure the absence of liver disease.*

R: Accordingly, we added the following sentences: “**Furthermore, transient hepatic elastography was also not performed because it was not accessible at the time of inclusion of the patients.** However, liver biopsy is not routinely performed in these patients in the real-life clinical practice, and **many centers (mainly in the primary care set) still do not have easy access to transient hepatic elastography.**”

*• I am not sure I understand the sentence “These patients represent the largest subgroup of heavy-drinkers on clinical practice”. I guess it depends on which healthcare setting one operates in?*

R: We clarified the sentence as following: “These patients represent the largest subgroup of heavy-drinkers on clinical practice **(particularly in Primary Care Clinics)**”
